# Sequencing paediatric antiretroviral therapy in the context of a public health approach

**DOI:** 10.7448/IAS.18.7.20265

**Published:** 2015-12-02

**Authors:** Ragna S Boerma, T Sonia Boender, Michael Boele van Hensbroek, Tobias F Rinke de Wit, Kim CE Sigaloff

**Affiliations:** 1 Amsterdam Institute for Global Health and Development and Department of Global Health, Academic Medical Center of the University of Amsterdam, The Netherlands; 2 Global Child Health Group, Emma Children's Hospital/Academic Medical Center of the University of Amsterdam, The Netherlands; 3 Division of Infectious Diseases, Department of Internal Medicine, Academic Medical Center, University of Amsterdam, The Netherlands

**Keywords:** paediatric HIV, antiretroviral therapy, HIV drug resistance, protease inhibitor, non-nucleoside reverse transcriptase inhibitor, low- and middle-income countries

## Abstract

**Introduction:**

As access to prevention of mother-to-child transmission (PMTCT) efforts has increased, the total number of children being born with HIV has significantly decreased. However, those children who do become infected after PMTCT failure are at particular risk of HIV drug resistance, selected by exposure to maternal or paediatric antiretroviral drugs used before, during or after birth. As a consequence, the response to antiretroviral therapy (ART) in these children may be compromised, particularly when non-nucleoside reverse transcriptase inhibitors (NNRTIs) are used as part of the first-line regimen. We review evidence guiding choices of first- and second-line ART.

**Discussion:**

Children generally respond relatively well to ART. Clinical trials show the superiority of protease inhibitor (PI)- over NNRTI-based treatment in young children, but observational reports of NNRTI-containing regimens are usually favourable as well. This is reassuring as national guidelines often still recommend the use of NNRTI-based treatment for PMTCT-unexposed young children, due to the higher costs of PIs. After failure of NNRTI-based, first-line treatment, the rate of acquired drug resistance is high, but HIV may well be suppressed by PIs in second-line ART. By contrast, there are currently no adequate alternatives in resource-limited settings (RLS) for children failing either first- or second-line, PI-containing regimens.

**Conclusions:**

Affordable salvage treatment options for children in RLS are urgently needed.

## Introduction

The treatment of HIV-1 in children is more challenging than treatment of adults and is associated with an increased risk of virological failure. Children are vulnerable to developing HIV drug resistance due to various reasons, such as variability in pharmacokinetics, limited paediatric treatment options and lack of adherence support [1]. Moreover, drug exposure as part of the prevention of mother-to-child transmission (PMTCT) can lead to pre-treatment drug resistance [2–4], thus diminishing the chance of treatment success.

Clinical trials have found that children under three years of age on protease inhibitor (PI)-based, antiretroviral therapy (ART) experience less virological failure and death than children on non-nucleoside reverse transcriptase inhibitor (NNRTI)-based regimens, both in PMTCT-exposed and -unexposed children [5–7]. The World Health Organization (WHO) therefore recommends all children below three years of age to receive a PI-based regimen [lopinavir/ritonavir (LPV/r)], regardless of history of PMTCT exposure [8]. Unfortunately, despite these recommendations, the use of PIs for young children in low- and middle-income countries (LMIC) in routine programmes is limited due to practical barriers. PIs are more costly than NNRTIs, and infant formulations were, until recently, only available as a liquid that requires refrigeration [7,9,10].

In this commentary, we will compare PI-based versus NNRTI-based, first-line ART for children, and also discuss feasible ART sequencing approaches in children.

## Discussion

**Figure 1:**
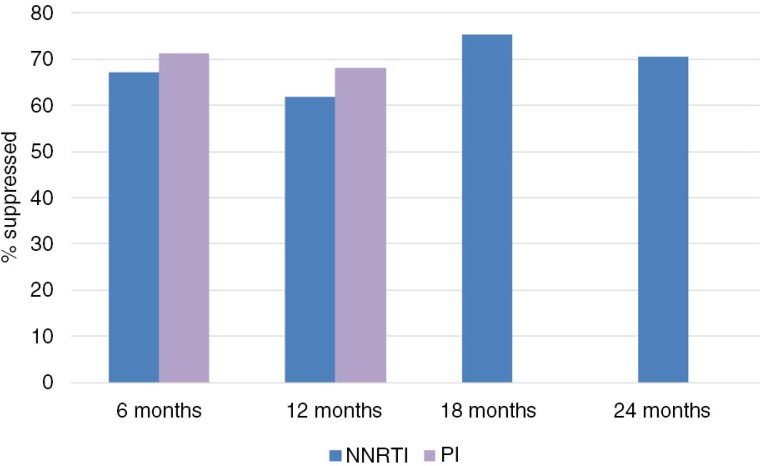
Summary estimates of virological suppression in children <3 years in LMIC, 6 to 24 months after first-line treatment initiation for NNRTI- and PI-treated children. Random effects meta-analysis was conducted using a Freeman–Tukey arcsine square root transformation to stabilize proportions. No virological suppression rates were available for PI-treated children after 18 and 24 months. NNRTI, non-nucleoside reverse transcriptase inhibitor; PI, protease inhibitor.

More than half of HIV-infected children who do not receive treatment are estimated to die before the age of two years [11]. ART dramatically reduces morbidity and mortality in HIV-infected children of all ages. Findings of previous systematic reviews are encouraging as up to 70 to 80% of children achieve virological suppression after 12 months of first-line treatment [12,13]. In young children under three years of age, data from clinical trials and observational studies in resource-limited settings (RLS) show that, on average, the HIV suppression rate is sustained around 60 to 70% up to 24 months after treatment initiation (Figure 1, Table 1).

**Table 1:** Studies reporting virological suppression rates in children <3 years on first-line ART 6–24 months after treatment initiation

Study	Median year of treatment initiation	Regimen	Total number of patients	Number of patients with viral suppression	% children with virological suppression	Time after treatment initiation
Lockman 2007^a^ [2]	2001	NNRTI-based	12	11	91.7	6 months
Lockman 2007^b^	2001	NNRTI-based	11	1	9.1	6 months
Puthanakit 2009 [14]	2004	NNRTI-based	25	14	56.0	6 months
Germanaud 2010 [15]	2007	NNRTI-based	68	43	63.2	6 months
Van Dijk 2011 [16]	2008	NNRTI-based	96	85	88.5	6 months
Cotton 2013 [17]	2006	PI-based	230	192	83.5	6 months
Romano Mazzotti 2009 [18]	Not reported	PI-based	56	21	37.5	6 months
Technau 2014 [19]	2006	PI-based	2612	1763	67.5	6 months
Lindsey 2014^a^ [20]	2008	NNRTI-based	116	86	74.1	6 months
Lindsey 2014^a^	2008	PI-based	124	112	90.3	6 months
Lindsey 2014^b^	2008	NNRTI-based	68	55	80.1	6 months
Lindsey 2014^b^	2008	PI-based	71	67	94.4	6 months
Meyers 2011 [21]	2006	PI-based	617	323	52.4	6 months
Lockman 2007^b^	2001	NNRTI-based	11	10	90.9	12 months
Lockman 2007^a^	2001	NNRTI-based	10	1	10.0	12 months
Jaspan 2008 [22]	2004	PI-based	85	60	70.6	12 months
Jaspan 2008	2004	NNRTI-based	115	47	40.9	12 months
Prendergast 2008 [23]	2004	PI-based	49	44	89.8	12 months
Puthanakit 2009	2004	NNRTI-based	24	19	79.2	12 months
Van Dijk 2011	2008	NNRTI-based	77	68	88.3	12 months
Romano Mazzotti 2009	Not reported	PI-based	56	30	53.6	12 months
Soeters 2014 [24]	2011	PI-based	118	61	51.7	12 months
Technau 2014	2006	PI-based	2165	1595	73.7	12 months
Puthanakit 2009	2004	NNRTI-based	19	16	84.2	18 months
Van Dijk 2011	2008	NNRTI-based	53	46	86.8	18 months
Kay2012 [25]	2007	NNRTI-based	34	19	55.9	18 months
Lockman 2007^b^	2001	NNRTI-based	9	1	11.1	24 months
Lockman 2007^a^	2001	NNRTI-based	11	9	81.8	24 months
Puthanakit 2009	2004	NNRTI-based	15	14	93.3	24 months
Van Dijk 2011	2008	NNRTI-based	27	21	77.8	24 months
Musiime 2014 [26]	2011	NNRTI-based	349	294	84.2	24 months

a PMTCT-unexposed cohort;

b PMTCT-exposed cohort.

NNRTI, non-nucleoside reverse transcriptase inhibitor; PI, protease inhibitor.

### NNRTI- versus PI-based, first-line ART

Based on data from clinical trials [5,6,27], the WHO has moved to recommending PI-based, first-line ART for all children below three years, regardless of previous PMTCT exposure. Comparison of trials and observational data reveals higher rates of virological suppression among children receiving PI-based regimens (Figure 1). However, data on PI-based, first-line treatment in children are still scarce, compared to NNRTI-based treatment, and most available PI data are from clinical trials with a relatively short follow-up period. The P1060 trial, a multicentre trial conducted in South Africa, Zimbabwe, Zambia, Malawi, Uganda, Tanzania and India, compared 288 children up to three years of age treated with AZT and 3TC combined with either NVP or LPV/r using the primary end point of treatment failure or discontinuation after 24 weeks. Both among PMTCT-exposed and -unexposed children, significantly more children reached the primary end point in the NVP group compared to the LPV/r group: 40.8 versus 19.3% (*p*<0.001) [5,6]. By contrast, a study performed in South Africa by Teasdale *et al*. [28] reported 27% virological failure among children after 24 weeks of first-line, PI-based treatment. The higher failure rate in this cohort may be because children received either ritonavir-boosted lopinavir (LPV/r) or full-dose ritonavir, which is associated with diminished virological response and the emergence of major protease mutations [29]. Programmatic data, as have to date been reported mainly from South Africa [21,30], will be very valuable in assessing whether the favourable virological suppression rates reported by trials can be achieved in routine ART programmes.

Most data on the effectiveness of NNRTI-based, first-line treatment are from programmatic settings. A retrospective cohort of 202 children starting NNRTI-based, first-line treatment in Thailand reported that 33 (16%) children had virological failure in the first year of treatment [31]. Children on NVP-based treatment were 3.3 times more likely to develop failure compared to children on EFV-based treatment. This study found no difference between young children with and without previous PMTCT exposure: 1 out of 4 and 4 out of 16 children, respectively, developed virological failure during the study period [31]. Two studies from sub-Saharan Africa show concordant results. Lowenthal *et al*. [32] describe a cohort study in Botswana with five years of follow-up including 804 children starting on EFV- or NVP-based, first-line treatment. The virological failure rate was 6.7% after one year, 10.2% after two years and 12.8% after five years of follow-up on EFV-based treatment, and 12.8, 19.8 and 25.1%, respectively, for NVP-based treatment [32]. In a Zambian cohort, 198 ART-naive and mostly PMTCT-unexposed children started either NVP- or EFV-based treatment. Six to twenty-four months after treatment initiation, the virological failure rate increased from 11.5 to 22.2% [16].

Interpretation of the differences between PI- and NNRTI-treated children is limited by the heterogeneity of studies in terms of design, study participants and setting. It is difficult to draw firm conclusions on the benefits of PI over NNRTI treatment in programmatic settings, especially in PMTCT-unexposed young children. However, results from randomized controlled trials have convincingly shown the superiority of PI- over NNRTI-based treatment [5,6], and PI-based treatment should be implemented for all HIV-infected children under three years of age, as recommended by the WHO [8]. The outcomes of observational studies reporting on programmatic data remain relevant, because the dispensation of PIs may be influenced by financial and logistical issues. LPV/r, currently the only PI combination available for children, is at least five times more expensive than EFV or NVP [33]. Recently, the United States Food and Drug Administration approved LPV/r in pellet form for paediatric usage, which, in contrast to the up-to-now only available LPV/r syrup, does not require refrigeration [10]. This is an important step towards increased access to PI treatment for children in LMIC.

### HIV-TB coinfection

Tuberculosis (TB) is one of the most common co-infections affecting children with HIV, and cotreatment occurs in up to one-third of children [21]. Comedication for TB adds significant complexity to the treatment of children who also require or are already receiving ART. For children on LPV/r-based regimens, guidelines suggest to add ritonavir to achieve the full therapeutic dose [8]. An alternative is to change to a triple NRTI regimen [34] or to substitute NVP for LPV/r [8]. Children on NVP- or EFV-based ART can usually continue the same regimen (ensuring that NVP dose is 200 mg/m^2^) or can also be changed to a triple NRTI regimen. These changes in the ART regimen, as well as simultaneous use of TB drugs, put children at risk of developing drug toxicity, virological failure [21] and HIV drug resistance [35].

### Development of resistance on first-line therapy

Virological failure is defined by the WHO as two consecutive measurements of plasma viral load >1000 cps/mL after at least six months of treatment [8]. However, WHO definitions have changed over time and studies have reported different virological cut-offs to define failure. A systematic review of resistance data in children from resource-poor settings found that 90% of those failing first-line regimens had at least one HIV drug-resistance mutation, with mutations increasing in frequency with duration of treatment [36]. This review included mostly cross-sectional studies and included children who were treated with suboptimal regimens.

More recent studies also show high rates of HIV drug resistance among children with treatment failure. In a study conducted in the Central African Republic, 83 and 85% of children on first-line therapy with a detectable viral load after 18 months had NRTI and NNRTI mutations, respectively. The most prevalent NRTI mutations were M184V (73%), T69D/N/S (17%), L74I/V (8%), K65R (8%) and Q151M (2%), and the most prevalent NNRTI mutations were Y181C (44%), K103H/N/S (39%), K101E/P (39%), G190A (30%) and A98G/S (19%) [37].

In Thai children treated with NVP- or EFV-containing therapy, NRTI mutations were found in 89% of children at the time of virological failure, with M184V/I (85%), K65R (11%) and K219Q/E (8%) being the most prevalent. NNRTI mutations were detected in 97% of the children, of which Y181C/I (58%), K103N (34%), G190S/A (18%) and V108I (13%), were most common [31].

It is clear from these studies that children who fail NNRTI-based, first-line regimens, generally report similarly high rates of NNRTI- and NRTI-associated mutations, with the Y181C and M184V mutations being among the most prevalent mutations within the respective drug classes. Accumulated NRTI resistance can have consequences for the construction of an effective, second-line, PI-based regimen, in which NRTIs are used as the backbone. This implies that a timely switch to second-line ART after failure is warranted, to prevent clinical consequences as well as the accumulation of drug resistance. Timely switching is, however, challenged by lack of virological monitoring in RLS. Reluctance of clinicians to change therapy in children, for whom limited drug options are available, may be an additional barrier.

In a European study, the development of both PI and NRTI resistance among children failing first-line, PI-based regimens was negligible [38]. In RLS, there are few reports of acquired protease mutations on first-line treatment. A recent South African study found that 8 out of 75 (10.7%) children with virological failure on a first-line PI had LPV/r mutations [39]. Within the NRTI drug class, the M184V and thymidine analogue mutations were found in seven out of eight and two out of eight children, respectively. Data among adults have shown that with intensified adherence support, viral load resuppression on PI-based ART is possible, despite drug resistance [40]. In this study, performed in Khayelitsha, South Africa, two-third of participants resuppressed within three months while remaining on PI-based regimens. The consequences of this study obviously extend to children receiving PIs; intensive adherence counselling should be offered before switching.

### Second-line ART

As per WHO recommendation, failure of an NNRTI-based regimen is followed by switching to a boosted PI plus two NRTIs. There are limited data about the response to second-line ART in children [41]. A recent study from Thailand reported on 111 children among whom the risk of virological failure 24 months after second-line initiation was 41% [42]. Children with longer duration of first-line ART were at higher risk of second-line failure. The latter suggests that continued first-line failure may have led to the accumulation of NRTI mutations, diminishing the response to subsequent second-line therapy. However, in the study's multivariate analysis, resistance to NRTIs did not appear as a risk factor for failure.

For children for whom a PI-based, first-line regimen has failed, NNRTIs remain the only new drug class that can be introduced. However, potential re-emergence of archived NNRTI mutations may limit the effectiveness of this ART sequencing approach. Moreover, NNRTIs have a much lower genetic barrier for resistance [43], and without the protection of an effective NRTI backbone (due to acquired resistance), NNRTI resistance will rapidly emerge. Recently, the first reports on the outcome of second-line NNRTI in children have been published. One small study from South Africa found that six months after regimen change, the proportion with virological failure was 75% (6 out of 8) in children receiving NNRTI-based second-line versus 20% (13 out of 66) in children on PI-based second-line [44]. A second study, again from South Africa, reported on 12 children who were switched to NNRTI-based therapy. Of these, 8 out of 12 (67%) did not achieve virological suppression [39]. Although these findings are based on a small number of children, it is apparent that NNRTI-based, second-line ART is not an optimal choice and is expected to have limited durability.

### Salvage options

Constructing third-line regimens using novel, robust drugs such as darunavir, raltegravir or dolutegravir, may be possible for children. Studies have demonstrated the efficacy of darunavir in heavily ART-experienced patients [45]. In a UK cohort, even in children with prolonged PI exposure, resistance to darunavir was rare [46]. Darunavir could therefore be an option after failure of first-line, LPV/r-based treatment in children above three years of age. Raltegravir is the first integrase inhibitor approved for paediatric usage (>4 weeks of age) and has been evaluated in the IMPAACT P1066 trial, showing virological suppression (<400 cps/mL) in approximately 80% of participants after 48 weeks of follow-up [47]. In adults, co-administration of rifampicin decreases raltegravir concentrations, thereby potentially limiting the efficacy of this drug in children with HIV-TB coinfection [48]. Dolutegravir, an integrase strand transfer inhibitor with a very favourable resistance profile, has to date only been approved in children >12 years of age. Results of two cohorts of the IMPAACT 1093 trial have been presented in an abstract form and showed virological suppression in 17 out of 23 treatment-experienced adolescents (aged 12 to 18 years) after 48 weeks of treatment with dolutegravir, and in 9 out of 11 treatment-experienced children (aged 6 to 12 years) after 24 weeks of treatment [49,50]. These newer antiretroviral agents, however, are currently unavailable in RLS. Substantial cost-reduction and/or generic production of these drugs are vital to ensure salvage options for children failing PI-based regimens.

## Conclusions

Despite the challenges of paediatric antiretroviral treatment, especially in RLS, studies have shown relatively high rates of virological suppression in children on first-line treatment. For young children, randomized controlled trials have shown the superiority of PI- over NNRTI-based treatment. Observational studies, however, also report favourable results of NNRTI-based, first-line treatment. This has important implications for settings in which PI treatment is unavailable due to logistic and financial barriers. Unquestionably, early initiation of treatment is vital and should be prioritized even if NNRTIs are the only obtainable drugs.

After NNRTI-based, first-line treatment failure, the rates of acquired drug resistance among children are strikingly high. However, these children are likely to still benefit from PIs in second-line. By contrast, the development of resistance mutations after failure of PI-based first-line is limited. If children do have continued failure on first-line LPV/r, the chances of resuppression after switching to second-line NNRTI are very low. Suitable formulations of additional PIs are urgently needed for children who fail either first- or second-line LPV/r. Darunavir boosted with ritonavir would be a suitable candidate, but it is not widely available. Newer antiretroviral agents including second-generation NNRTIs and integrase inhibitors should also be evaluated. The future of an increasing number of children will depend on the availability of these salvage medications. To make these regimens accessible on a global scale, low-cost generic drugs or major price reductions of patented versions are necessary.
